# Regulation of neurite growth by tumour necrosis superfamily member RANKL

**DOI:** 10.1098/rsob.120150

**Published:** 2013-01

**Authors:** Humberto Gutierrez, Lilian Kisiswa, Gerard W. O'Keeffe, Matthew J. Smithen, Sean Wyatt, Alun M. Davies

**Affiliations:** Cardiff School of Biosciences, Cardiff University, Cardiff Wales CF10 3AX, UK

**Keywords:** sympathetic neuron, sensory neuron, neurite growth, RANKL, RANK, development

## Abstract

RANKL (receptor-activator of NF-κB ligand, TNFSF11) is a member of the TNF superfamily that regulates bone remodelling and the development of the thymus, lymph nodes and mammary glands. While RANKL and its membrane bound receptor RANK (TNFRSF11A) are expressed in the adult central nervous system and have been implicated in thermoregulation, the potential function of RANK signalling in the developing nervous system remains unexplored. Here, we show that RANK is expressed by sympathetic and sensory neurons of the developing mouse peripheral nervous system and that activating RANK signalling in these neurons during perinatal development by either treating cultured neurons with soluble RANKL or overexpressing RANK in the neurons inhibited neurotrophin-promoted neurite growth without affecting neurotrophin-promoted neuronal survival. RANKL is expressed in tissues innervated by these neurons, and studies in compartment cultures demonstrated that RANKL is capable of acting directly on neurites to inhibit growth locally. Enhancing RANK signalling in cultured neurons resulted in NF-κB activation and phosphorylation of the p65 NF-κB subunit on serine 536. Transfecting neurons with a series of mutated signalling proteins showed that NF-κB activation and p65 phosphorylation occurred by an IKKβ-dependent mechanism and that blockade of this signalling pathway prevented neurite growth inhibition by RANKL. These findings reveal that RANKL is a novel negative regulator of neurite growth from developing PNS neurons and that it exerts its effects by IKKβ-dependent activation of NF-κB.

## 2. Introduction

The growth and guidance of axons in the developing nervous system is regulated by numerous extracellular signals that either promote or inhibit axon extension and influence axon trajectory by acting as either attractants or repellents at the growth cone [1]. The sympathetic neurons of the developing mouse superior cervical ganglion (SCG) are an extensively studied, experimentally tractable population of neurons for investigating the regulation of axonal growth and target field innervation in the peripheral nervous system [2,3]. A variety of secreted signalling proteins have been shown to promote sympathetic axon growth, including nerve growth factor (NGF), neurotrophin-3 (NT**^_^**3), glial cell-derived neurotrophic factor (GDNF), artemin and hepatocyte growth factor (HGF). Of these, the most extensively studied and best understood is NGF, which is produced in the tissues innervated by these neurons, and which promotes the growth and branching of sympathetic axons within these tissues [4]. Target-derived NGF also has a well-established role in promoting and regulating sympathetic neuron survival during development [5]. In addition to factors that promote growth and branching, brain-derived neurotrophic factor (BDNF) has been shown to inhibit sympathetic axon growth [6] and to play a role in the axon pruning that establishes regional differences in innervation density [7].

To identify additional candidate extracellular regulators of sympathetic axonal growth, we carried out a QPCR screen for the expression of known families of receptors in the developing SCG at stages of development from initial axon extension to establishment of target field innervation. Among the receptor transcripts expressed at high levels was that for RANK (receptor-activator of NF-κB, TNFRSF11A), a member of the tumour necrosis factor (TNF) receptor superfamily. The 19 members of the TNF superfamily of ligands are best understood for their many critical functions in the development and function of the immune system, and are increasingly recognized as having diverse roles in other tissues and organs [8]. These cytokines are type II transmembrane glycoproteins that are active both as membrane-bound ligands and as soluble ligands following cleavage from the cell membrane by the action of specific metalloproteinases. TNFSF ligands bind to one or more members of the TNF receptor superfamily, which are classified into three groups: those containing a proapoptotic death domain, those lacking a death domain and a group of decoy receptors that do not transmit any signal [9].

RANK lacks a death domain and interacts with adaptors of the TRAF family to modulate several signalling pathways, including the NF-κB signalling pathway [10]. RANK and its ligand RANKL play essential roles in the immune system, where they regulate dendritic cell survival and maturation, lymphocyte differentiation, and T-cell responses [11]. RANK signalling is also a crucial regulator of bone turnover by controlling osteoclast differentiation and activation [12], and is important for the formation of lobulo-alveolar mammary structures during pregnancy [13]. Osteoprotegerin (OPG), a decoy receptor for RANKL, competes with RANK for binding to RANKL, thereby negatively regulating RANK signalling [14]. RANKL and RANK are also expressed in the central nervous system [15], where they have been implicated in thermoregulation and the central fever response to inflammation [16]. However, the potential role of RANK signalling in neuronal development has not been explored. Here we report a novel function for RANKL as a potent negative regulator of neurotrophin-promoted neurite growth, but not survival, in sympathetic and sensory neurons of developing peripheral neurons, and show that this effect is mediated by a mechanism involving canonical NF-κB signalling.

## 3. Results

### 3.1. RANK is expressed in developing superior cervical ganglion neurons

**Figure 1.**
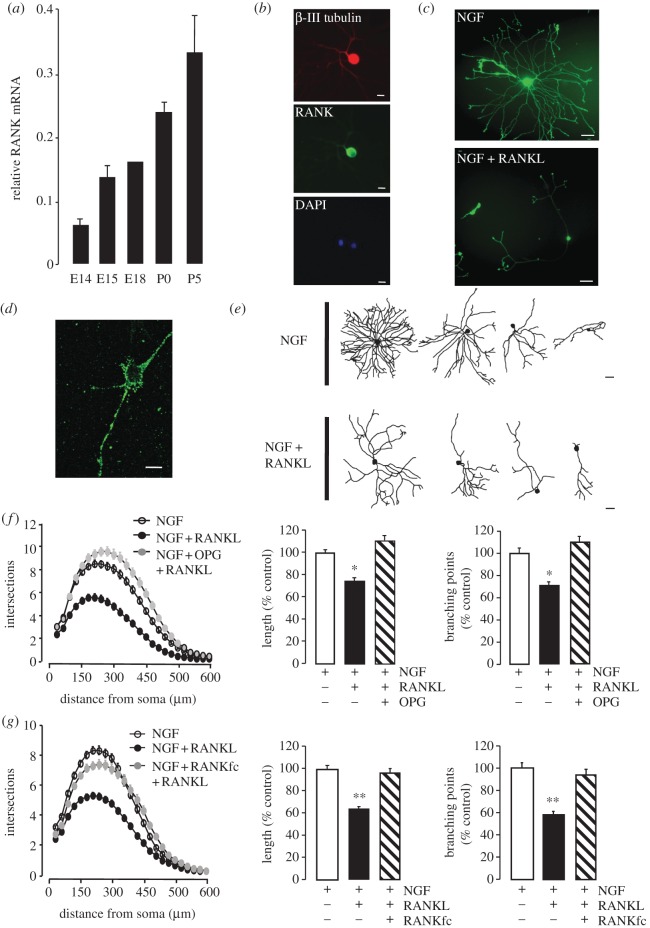
RANK is expressed in developing SCG neurons and its activation inhibits NGF-promoted neurite growth. (*a*) Levels of RANK mRNA relative to the reference mRNAs, GAPDH and SDHA, in RNA extracted from SCG at ages ranging from E14 to P5. The mean ± s.e.m. of data obtained from four separate sets of ganglia at each age are shown. (*b*) Photomicrographs of a P0 SCG neuron immunostained for RANK and β-III tubulin after 24 h incubation in medium containing 10 ng ml^–1^ NGF. Scale bar = 10 μm. A non-neuronal cell whose nucleus, along with the neuronal nucleus, is stained with the nuclear marker DAPI was unlabelled by the anti-RANK antibody. (*c*) Representative photomicrographs of P0 SCG neurons labelled with calcein AM after 24 h incubation in medium containing either 10 ng ml^−1^ NGF or NGF plus 10 ng ml^–1^ recombinant RANKL. (*d*) Confocal optical section through a neuron cell body. Scale bar = 10 μm. (*e*) Representative camera lucida drawings of neurons illustrating the range of morphologies of P0 SCG neurons after 24 h incubation in medium containing either NGF or NGF plus RANKL. The neurons illustrated correspond to percentiles 25, 50, 75 and 100 of the sampled populations in terms of total neurite length. Scale bar = 20 μm*.* (*f,g*) Sholl profiles, total length and number of branching points in the neurite arbours of P0 SCG neurons incubated for 24 h in medium containing 10 ng ml^−1^ NGF alone, NGF plus 10 ng ml^–1^ RANKL or NGF plus RANKL plus either (*f*) 10 ng ml^−1^ OPG or (*g*) 10 ng ml^–1^ RANKfc. To facilitate comparison of separate experiments, the length and branching point data are expressed as a percentage of the NGF values. Mean ± s.e.m. are shown, **p < 0.001, statistical comparison with NGF data. The non-normalized neurite length and branching datasets for all experiments carried out are provided in electronic supplementary material, spreadsheet S1.

To identify members of the TNF superfamily that may play a role in regulating axon growth, we carried out a real-time PCR screen for expression of the receptors for this superfamily in the mouse SCG at stages throughout embryonic and early post-natal development when axons are growing to and ramifying within their targets. This screen revealed prominent expression of RANK mRNA, with a sixfold increase in the level of expression between E14 and P5 relative to the reference mRNAs encoding the housekeeping proteins glyceraldehyde-3-phosphate dehydrogenase (GAPDH) and succinate dehydrogenase (SDHA; figure 1*a*). This spans the period of development from neurogenesis to the establishment of target field innervation [3]. To confirm expression of RANK protein and determine which cells express RANK protein, we used immunocytochemistry to localize RANK in dissociated cell cultures established from the SCG of newborn mice. These studies showed that the cell bodies and processes of all neurons (positively identified with an anti-βIII tubulin antibody) were labelled by a specific anti-RANK antibody, and that non-neuronal cells were unlabelled by this antibody (figure 1*b*). High-power confocal optical sections through the cell body showed predominantly membrane labelling (figure 1*d*). No cells were labelled when primary antibodies were omitted from the protocol (not shown).

### 3.2. RANKL impairs nerve growth factor-promoted neurite growth from developing superior cervical ganglion neurons

Having found especially high levels of RANK mRNA in the SCG during the stages of development when SCG axons are growing and branching in their targets and the neurons are additionally dependent on target-derived signals such as NGF for their survival, we investigated whether RANK signalling plays a role in regulating axonal growth and/or neuronal survival. To do this, we studied the effect of purified recombinant RANKL on neurite growth and neuronal survival in low-density dissociated cultures of newborn (P0) SCG neurons. In the absence of NGF, the great majority of neurons died within 24 h of plating, and RANKL neither enhanced survival nor promoted neurite growth from the small number of neurons that had not undergone apoptosis by 24 h (data not shown). Because RANKL alone had no obvious effect on SCG neurons, we investigated whether it modulates the effects of NGF on survival and neurite growth. In NGF-supplemented cultures, more than 80 per cent of P0 SCG neurons survived and elaborated extensive neurite arbours within 24 h of plating. The addition of RANKL to NGF-supplemented cultures had no significant effect on neuronal survival (data not shown), but markedly reduced the size and complexity of neurite arbours (figure 1*c,e*). This obvious reduction in neurite growth was confirmed by quantification of total neurite length and number of branch points, and by Sholl analysis, which provides a graphical illustration of neurite length and branching with distance from the cell body (figure 1*f,g*). To test the specificity of RANKL–RANK interaction in RANKL suppression of NGF-promoted neurite growth, we studied the effects of specific competitors of RANKL–RANK interaction. Both the naturally occurring decoy receptor OPG (figure 1*f*) and a recombinant soluble RANKfc chimeric protein (figure 1*g*) completely prevented the inhibitory effect of RANKL on NGF-promoted neurite growth. These results suggest that binding of RANKL to RANK triggers a neurite growth inhibitory signal in developing sympathetic neurons.

### 3.3. RANK signalling impairs nerve growth factor-promoted neurite growth from superior cervical ganglion neurons over an extended period of development

**Figure 2.**
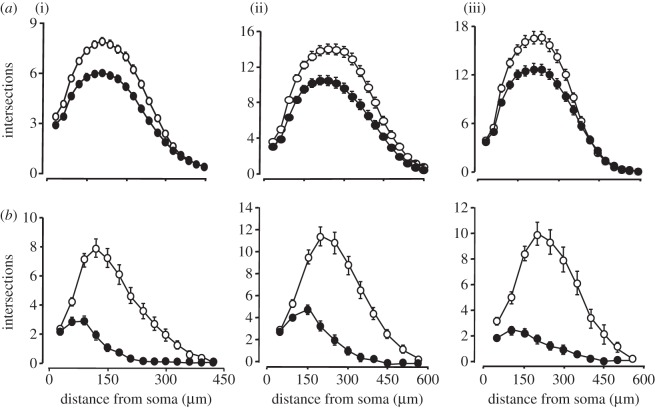
Recombinant RANKL and RANK overexpression impair NGF-promoted neurite growth from SCG neurons over an extended period of development. (*a*) Sholl profiles of SCG neurons isolated from (i) P0, (ii) P5 and (iii) P10 mice after 24 h incubation with either 10 ng ml^−1^ NGF (unfilled circles) or NGF plus 10 ng ml^−1^ RANKL (filled circles). (*b*) Sholl profiles of SCG neurons isolated from (i) P0, (ii) P5 and (iii) P10 mice that were incubated for 24 h in medium containing 10 ng ml^−1^ NGF after transfection with a YFP expression plasmid together with either an empty control plasmid (control, unfilled circles) or a YFP expression plasmid together a plasmid expressing RANK (filled circles). Means ± s.e.m. of typical experiments are shown at each age. The neurite length and branching datasets for all experiments carried out are provided in electronic supplementary material, spreadsheet S2.

To determine whether the inhibitory effect of RANK signalling on NGF-promoted neurite growth is restricted to a particular phase of development, we studied the effects of RANKL on neurite growth from SCG neurons cultured at stages throughout post-natal development. Sholl analysis revealed that RANKL reduced the size and complexity of the neurite arbours of SCG neurons grown with NGF from P0 to at least P10 (figure 2*a*). We additionally studied the effects of enhancing RANK signalling in SCG neurons by overexpressing RANK in these neurons by transfecting them with a plasmid that expresses RANK. Sholl analysis showed that neurite arbours of neurons overexpressing RANK were markedly smaller and less branched than those of control-transfected neurons in NGF-supplemented medium over this period of development (figure 2*b*). These results demonstrate that enhancing RANK signalling impairs NGF-promoted neurite growth from SCG neurons over an extended period of post-natal development.

### 3.4. RANKL is expressed in targets and acts locally on sympathetic axons to inhibit growth

**Figure 3.**
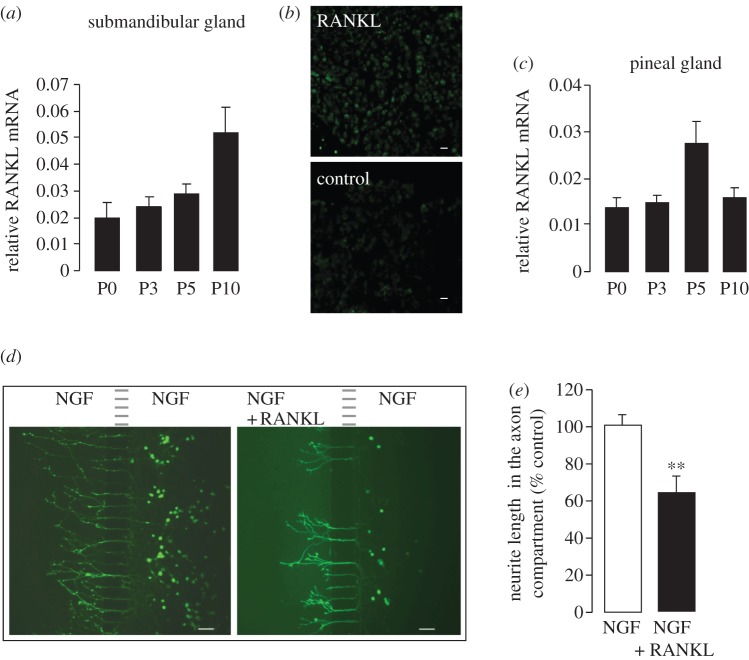
RANKL is expressed in sympathetic targets and acts directly on sympathetic axons to suppress NGF-promoted growth. (*a*) Levels of RANKL mRNA relative to GAPDH and SDHA mRNAs in RNA extracted from submandibular gland at ages ranging from P0 to P10. The mean ± s.e.m. of data obtained from four separate sets of glands at each age are shown. (*b*) Immunohistochemical localization of RANKL in a section through the submandibular gland at P0. A section incubated with secondary antibody alone (control) is also shown. Scale bar = 50 μm. (*c*) Levels of RANKL mRNA relative to GAPDH and SDHA mRNAs in RNA extracted from pineal gland at ages ranging from P0 to P10. The mean ± s.e.m. of data obtained from four separate sets of glands at each age are shown. (*d*) Representative images of calcein-AM-labelled P0 SCG neurons cultured for 24 h in a two-compartment microfluidic device containing 10 ng ml^−1^ NGF in both compartments (left) or NGF in both compartments plus 10 ng ml^−1^ RANKL in the axon compartment (right). Scale bar = 50 μm. (*e*) Bar chart of mean axon length of neurons projecting axons into the axon compartment in cultures containing only NGF in both compartments or NGF in both compartments plus RANKL in the axon compartment. The results are expressed as a percentage of axon length in cultures containing NGF alone in both compartments (mean ± s.e.m. are shown, **p < 0.001, statistical comparison with cultures containing NGF alone in both compartments). The non-normalized datasets of all experiments carried out are provided in electronic supplementary material, spreadsheet S3.

To investigate the developmental context in which RANKL might influence the SCG neuron development and modulate the innervation of sympathetic targets, we asked whether RANKL is expressed in SCG target tissues at stages throughout the period of development when sympathetic axons are growing and branching within these targets. To this end, we quantified RANKL mRNA levels in two anatomically discrete and easily dissectable target tissues: the submandibular gland and the pineal gland. During the immediate post-natal period, there was a gradual increase in the expression of RANKL mRNA in the submandibular gland, reaching a peak by P10 (figure 3*a*), and a sustained expression of RANKL mRNA in the pineal gland, although a transient peak was evident at P5 (figure 3*c*). Histological sections through the submandibular gland showed RANKL immunoreactivity in the parenchyma of this gland (figure 3*b*). These findings raise the possibility that RANKL could act on SCG axons within their target tissues.

To assess whether RANKL is capable of exerting a local inhibitory effect on the growth of sympathetic axons, we cultured SCG neurons in microfluidic devices in which the cell bodies and neurites are grown in separate compartments, permitting independent experimental manipulation of the environment in each compartment. P0 SCG neurons were seeded into one compartment (the cell body compartment) that was supplemented with NGF to maintain neuronal viability. The other compartment (the neurite compartment) received either NGF alone or NGF plus RANKL. After 24 h incubation, the neurite arbours and cell bodies of neurons that extended neurites into the neurite compartment were labelled with calcein AM added to the neurite compartment. Average neurite length per labelled soma was estimated by counting intersections between all neurites and a series of equally spaced parallel lines digitally traced in the neuritic compartment. Addition of RANKL to the neurite compartment caused a highly significant decrease in neurite length compared with neurites growing with NGF alone (figure 3*d,e*). Taken together, these results show that RANKL is expressed in sympathetic targets, and can potentially act on sympathetic axons to restrict their extension and branching locally.

### 3.5. RANKL/RANK signalling enhances NF-κB transcriptional activity in superior cervical ganglion neurons

**Figure 4.**
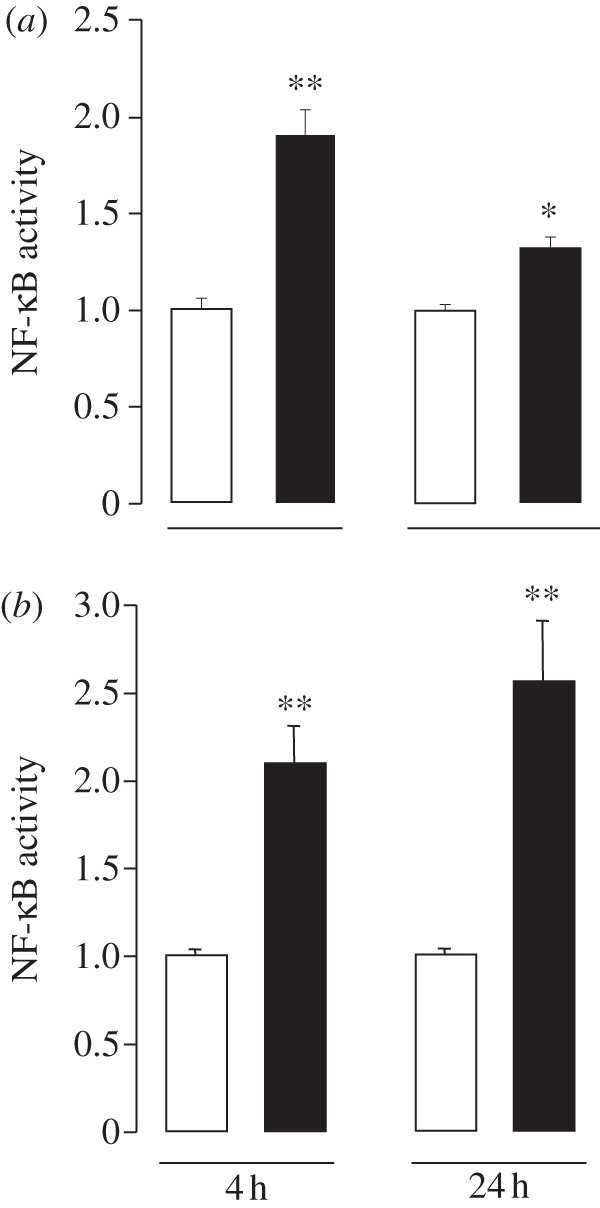
Recombinant RANKL and RANK overexpression activate NF-κB in SCG neurons. (*a*) NF-κB reporter signal in P0 SCG neurons incubated for 4 and 24 h in medium containing 10 ng ml^−1^ NGF alone (unfilled bars) and 10 ng ml^–1^ NGF plus 10 ng ml^–1^ RANKL (filled bars). (*b*) NF-κB reporter signal in P0 SCG neurons transfected with either a RANK (filled bars) expression plasmid or a control (unfilled bars) empty plasmid and incubated for 4 and 24 h in medium containing 10 ng ml^–1^ NGF. The data are normalized to the respective control signal. Mean ± s.e.m. are shown, **p < 0.001, statistical comparison with the respective controls. The non-normalized datasets of these experiments are provided in electronic supplementary material, spreadsheet S4.

Because the NF-κB transcription factor complex plays a role in mediating several of the cellular responses to members of the TNFSF, including RANK [9,10], and NF-κB signalling has been implicated in the regulation of neurite growth [17], we investigated the potential role of NF-κB in mediating the neurite growth inhibitory effect of RANKL/RANK signalling. First, we tested whether RANKL and RANK signalling enhance NF-κB transcriptional activity in SCG neurons. The level of NF-κB transcriptional activity was measured by transfecting P0 SCG neurons with a reporter construct in which GFP expression is under the control of a series of κB regulatory elements [18]. The neurons were then plated in NGF-containing medium with and without RANKL. The NF-κB reporter signal was quantified in the transfected neurons after 4 and 24 h in culture. RANKL stimulation resulted in significant increases in the reporter signal at both time points, but especially after 4 h of RANKL stimulation (figure 4*a*). Enhancing RANK signalling by overexpressing RANK in the neurons by transfecting them with a RANK expression plasmid also enhanced the NF-κB reporter signal, although NF-κB transcriptional activity was sustained in RANK transfected neurons throughout the 24 h culture period (figure 4*b*). These results show that RANK signalling enhances NF-κB transcriptional activity in developing SCG neurons.

### 3.6. Neurite growth inhibition by RANK signalling requires IKKβ- (but not IKKα-) mediated NF-κB activation

To assess the role of NF-κB in neurite inhibition brought about by RANK signalling, we studied the magnitude of NGF-promoted neurite growth from P0 SCG neurons co-transfected with the RANK expression plasmid and plasmids expressing mutated proteins that interfere with key steps in NF-κB signalling and activation. RANK overexpression was used to dissect steps in NF-κB signalling required for neurite growth inhibition because this gives prolonged activation of NF-κB and has a proportionally greater effect on neurite growth inhibition, so the effects of manipulating different steps in NF-κB signalling on the magnitude of neurite growth inhibition are easier to observe.

**Figure 5.**
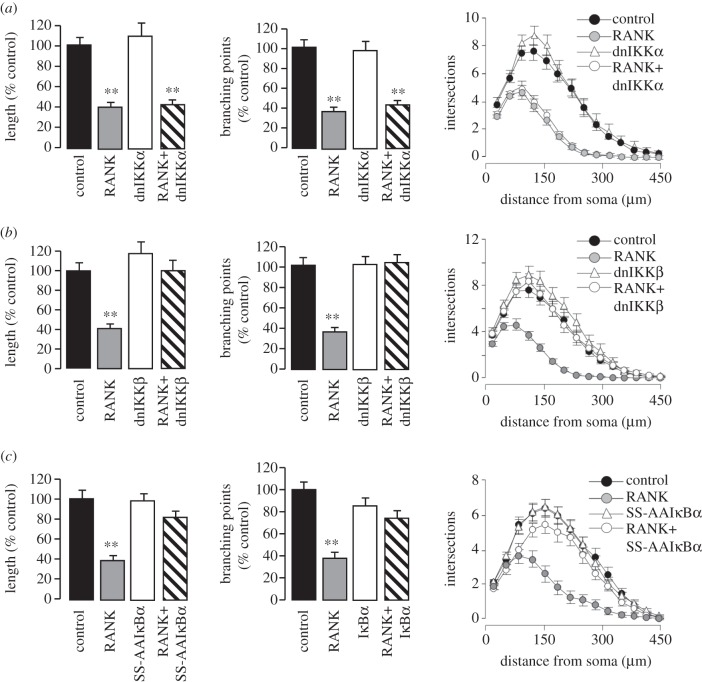
Neurite growth inhibition associated with RANK overexpression requires IKKβ- (but not IKKα-) mediated NF-κB activation. (*a–c*) Bar charts of neurite length and branch point number, and Sholl profiles, of P0 SCG neurons after 24 h incubation in medium containing 10 ng ml^−1^ NGF following co-transfection with a YFP plasmid together with plasmids expressing: (*a*) RANK, dominant-negative IKKα (dnIKKα) and RANK plus dnIKKα; (*b*) RANK, dominant-negative IKKβ (dnIKKβ) and RANK plus dnIKKβ; and (*c*) RANK, S32A/S36A IκBα and RANK plus IκBα. To facilitate comparison of data from all experiments, the length and branch point data are expressed as a percentage of control-transfected values. Means ± s.e.m. are shown, **p < 0.001, statistical comparison with the respective controls. The non-normalized neurite length and branching datasets for all experimental results are provided in electronic supplementary material, spreadsheet S5.

In the canonical NF-κB signalling pathway, the NF-κB inhibitory protein IκBα is phosphorylated by the IKKβ subunit of an IκB kinase (IKK) complex, resulting in its ubiquitination and proteasome-mediated degradation. Following IκBα degradation the p65/p50 NF-κB heterodimer, which is normally held in the cytosol by its interaction with IκBα, translocates to the nucleus, where it binds to κB elements in the promoter and enhancer regions of responsive genes. In the non-canonical NF-κB signalling pathway, the p100 subunit of the p100/RelB NF-κB heterodimer is phosphorylated by the IKKα subunit of the IKK complex, resulting in ubiquitination and proteosome-dependent processing of p100 to p52, followed by translocation of the resulting p52/RelB heterodimer to the nucleus [19]. Expression of a dominant-negative IKKα protein (K44A-IKKα) in SCG neurons had no effect on the magnitude of NGF-promoted neurite growth compared with control-transfected neurons and did not affect the suppression of NGF-promoted neurite growth by RANK overexpression (figure 5*a*). This suggests that IKKα plays no role in the suppression of NGF-promoted neurite growth by RANK signalling. In marked contrast, expression of dominant-negative IKKβ protein (K44A-IKKβ) in SCG neurons completely reversed the suppression of NGF-promoted neurite growth caused by RANK overexpression, while having no effect on NGF-promoted neurite growth when expressed alone (figure 5*b*). This suggests that IKKβ (but not IKKα) is required for RANK-induced suppression of NGF-promoted neurite growth.

To test further the requirement for canonical NF-κB signalling in RANK-mediated neurite growth inhibition, we transfected P0 SCG neurons with a plasmid that expresses a mutated IκBα protein that has serine-to-alanine substitutions at residues phosphorylated by IKKβ. As previously reported [20], this S32A/S36A IκBα mutant affected neither NGF-promoted neurite growth (figure 5*c*) nor NGF-promoted survival (not shown). However, when the S32A/S36A IκBα plasmid was co-transfected with the RANK plasmid, it completely prevented the inhibitory effect of the latter on NGF-promoted neurite growth (figure 5*c*), suggesting that serine phosphorylation of IκBα (and hence canonical activation of NF-κB) is critical for neurite growth inhibition associated with RANK overexpression.

### 3.7. Phosphorylation of p65 at serine 536 is required for neurite growth inhibition by RANK signalling

**Figure 6.**
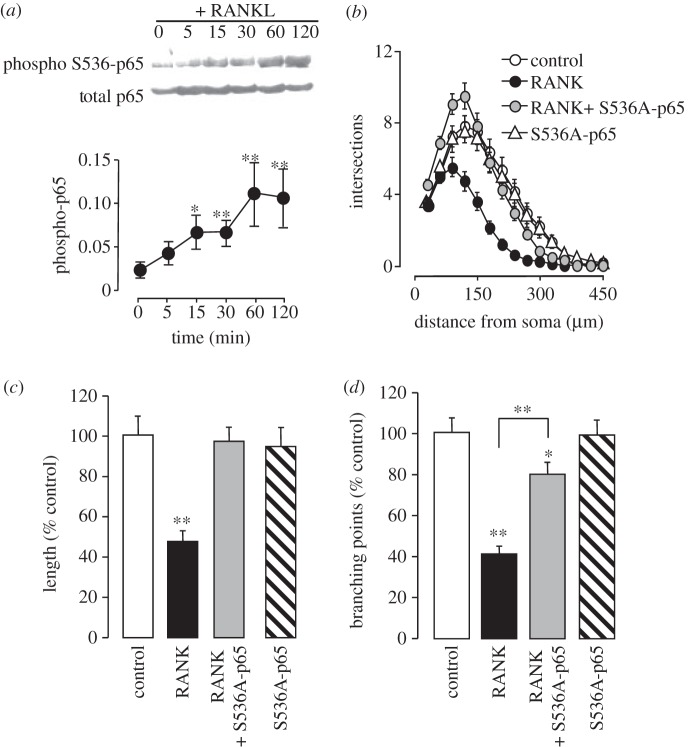
Phosphorylation of the p65 NF-κB subunit at ser536 is required for RANK-mediated inhibition of neurite growth. (*a*) Representative Western blot of phospho-S536-p65 and total p65, and graph of time course of p65 phosphorylation in cultured SCG neurons following stimulation with 10 ng ml^−1^ RANKL after 12 h pre-incubation with 10 ng ml^−1^ NGF. Each data point represents the mean ± s.e.m. of six separate Western blot assays. (*b*) Sholl profiles and bar charts of (*c*) total neurite length and (*d*) branch point number of the neurite arbours of P0 SCG neurons after 24 h incubation in medium containing 10 ng ml^−1^ NGF following co-transfection with a YFP plasmid together with either an empty control plasmid or plasmids expressing RANK, S536A-p65 or RANK plus S536A-p65. The length and branch point data are expressed as a percentage of data from control-transfected neurons. Means ± s.e.m. of typical experiments are shown (50–90 neurons per condition, **p < 0.001, statistical comparison with the respective controls). Sholl plots from representative experiments are shown. The non-normalized neurite length and branching datasets for all experiments carried out are provided in electronic supplementary material, spreadsheet S6.

We have recently shown that activated NF-κB can either promote or inhibit neurite growth depending on the activation mechanism and the phosphorylation status of the p65 NF-κB subunit [20]. In addition to phosphorylating IκBα on serines 32 and 36, activated IKKβ has been shown to phosphorylate p65 on serine 536 [21–24], resulting in a neurite growth-inhibiting form of the activated p65/p50 heterodimer [20]. To ascertain whether treatment of SCG neurons with RANKL leads to phosphorylation of p65 on S536, Western analysis was carried on lysates of P0 SCG neurons that had been treated with RANKL for different times following overnight pre-incubation with NGF. This analysis revealed a significant increase in the level of phospho-S536-p65 relative to total p65 within 15 min of RANKL treatment, followed by a further gradual rise in the level of phospho-S536-p65 over 60 min (figure 6*a*). To test whether p65 phosphorylation on S536 is required for neurite growth inhibition associated with RANK overexpression, we studied the effect of transfecting P0 SCG neurons with a plasmid that expresses a S536A-p65 mutant, which prevents phosphorylation at S536 without interfering with transcriptional activity [21,25,26]. This S536A-p65 mutant did not significantly affect NGF-promoted neurite growth when expressed alone, but prevented the neurite growth inhibitory effect of RANK overexpression (figure 6*b–d*). This suggests that p65 phosphorylation at serine 536 is required for neurite growth inhibition induced by RANK signalling.

### 3.8. RANK signalling suppresses neurite growth from developing sensory neurons

**Figure 7.**
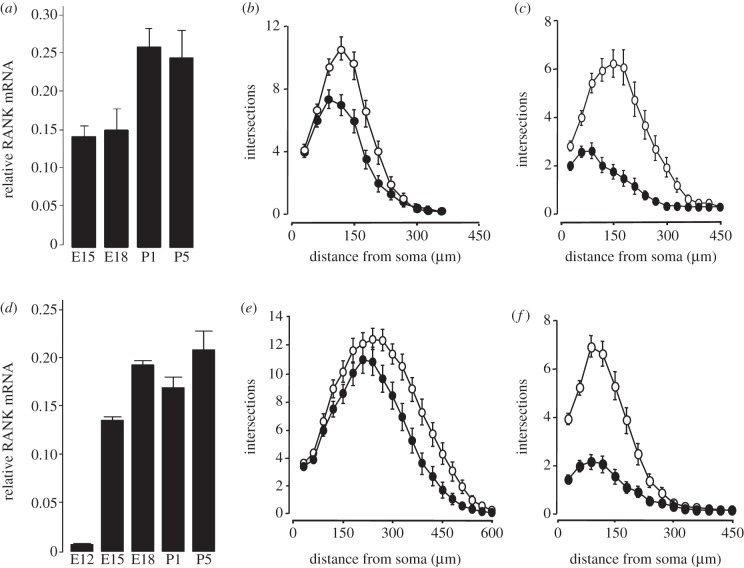
RANK is expressed in developing trigeminal and nodose neurons, and its activation inhibits neurotrophin-promoted neurite growth. (*a,d*) Relative levels of RANK mRNAs in total RNA extracted from (*a*) nodose and (*d*) trigeminal ganglia at the ages indicated. The levels of these mRNAs were normalized to the level of the reference GAPDH and SDHA mRNAs at each age. The mean ± s.e.m. of data obtained from at least three separate sets of dissected ganglia at each age are shown. (*b,c*) Sholl profiles of the neurite arbours of P1 nodose neurons incubated for 24 h with 10 ng ml^−1^ BDNF and additionally (*b*) treated with 10 ng ml^−1^ RANKL or (*c*) transfected with either an empty control plasmid or a RANK expression plasmid. (*e,f*) Sholl profiles of the neurite arbours of P1 trigeminal neurons incubated for 24 h with 10 ng ml^−1^ NGF and additionally (*e*) treated with 10 ng ml^−1^ RANKL or (*f*) transfected with either an empty control plasmid or a RANK expression plasmid. Sholl plots from representative experiments are shown. The datasets of all experiments carried out are provided in electronic supplementary material, spreadsheet S7. (*b,e*) Unfilled circles, control; filled circles, RANK. (*c,f*) Unfilled circles, control; filled circles, RANKL.

To investigate whether RANK signalling exerts an inhibitory effect on neurite growth from other populations of developing neurons, we studied two other experimentally tractable populations of developing peripheral neurons: the NGF-dependent neurons of the trigeminal ganglion and the BDNF-dependent neurons of the nodose ganglion. QPCR showed that both ganglia express RANK mRNA, and revealed an increase in the relative level of RANK mRNA throughout embryonic and post-natal development (figure 7*a,d*). Treatment with RANKL significantly reduced BDNF-promoted neurite growth from P1 nodose neurons (figure 7*b*) and significantly reduced NGF-promoted neurite growth from P1 trigeminal neurons (figure 7*e*). In these cultures, more than 80 per cent of the neurons survived with the respective neurotrophin and the addition of RANKL to the medium did not affect survival (not shown). Likewise, transfecting nodose and trigeminal neurons with the RANK expression plasmid markedly reduced BDNF-promoted (figure 7*c*) and NGF-promoted (figure 7*f*) neurite growth compared with control-transfected neurons, again without affecting survival (not shown). These results suggest that RANK signalling negatively regulates neurotrophin-promoted neurite growth in several populations of developing neurons without affecting neurotrophin-promoted survival. Furthermore, expression of either K44A-IKKβ or S536A-p65 (but not K44A-IKKα) in sensory neurons completely prevented suppression of neurotrophin-promoted neurite growth by RANK overexpression (data not shown), demonstrating the general involvement of IKKβ-dependent NF-κB canonical signalling in RANK-mediated neurite growth inhibition in both sympathetic and sensory neurons.

## 4. Discussion

We have discovered that RANKL is a potent negative regulator of axon growth and branching in the developing peripheral nervous system. Soluble RANKL and activation of RANK signalling by overexpressing RANK markedly reduced the size and complexity of the neurite arbours of perinatal SCG sympathetic neurons and trigeminal sensory neurons cultured with NGF, and nodose sensory neurons cultured with BDNF. Neither RANKL nor RANK overexpression affected the survival of these neurons with neurotrophins, indicating that RANK signalling has a selective effect on neurotrophin-promoted axonal growth and branching, but not survival. Detailed developmental studies on SCG neurons indicated that RANKL and RANK overexpression inhibit NGF-promoted neurite growth over an extended period of post-natal development, from P0 to at least P10. Throughout this period of development, the axons of SCG neurons are growing and branching within their target tissues under the influence of target-derived NGF. Like NGF, RANKL is expressed in SCG target tissues over this period of development. In two anatomically discrete SCG target organs, the pineal and submandubular glands, RANKL mRNA was clearly detected throughout early post-natal development. Moreover, immunohistochemistry confirmed the presence of RANKL protein in cells of the target field. Thus, axon growth-promoting and growth-inhibiting factors are expressed in the same tissues. Studies using compartment cultures have clearly shown that NGF acts directly on axons to promote the growth of only those axon branches exposed to it [27]. Likewise, using compartment cultures, we also show that RANKL acts directly on axons to inhibit their growth. Thus, it is possible that the precise cellular disposition of NGF and RANKL within tissues might fine-tune the growth and branching, and hence the specific distribution of axons and their terminals within these tissues, by a combination of antagonistic growth-promoting and growth-inhibitory influences. RANK-deficient mice are embryonic lethal [11]; thus a conditional knockout approach will be necessary in future work to clarify the *in vivo* role of RANK signalling in regulating tissue innervation during post-natal development.

RANK lacks a death domain and interacts with adaptors of the TRAF family to modulate several signalling pathways, including the NF-κB signalling pathway [10]. NF-κB signalling has been shown to regulate axon and dendrite growth in the developing peripheral and central nervous system [17]. Importantly, IKKβ-dependent NF-κB activation, with its attendant phosphorylation of the p65 NF-κB subunit on serine 536, exerts a strong inhibitory influence on neurite growth, whereas NF-κB activation via an alternative tyrosine kinase-dependent mechanism promotes neurite growth [20]. Here we demonstrate that RANKL and RANK signalling promote IKKβ-dependent NF-κB activation in both SCG and nodose neurons, and cause a rapid increase in the levels of phospho-S536-p65. Furthermore, transfecting these neurons with plasmids that express mutated versions of NF-κB signalling proteins has demonstrated that IKKβ-dependent NF-κB activation is responsible for RANK-mediated neurite growth inhibition. Expression of a dominant-negative K44A-IKKβ mutant, but not a dominant-negative K44A-IKKα mutant, prevents RANK-mediated neurite growth inhibition, as does a S32A/S36A IκBα mutant, which cannot be phosphorylated by IKKβ and hence cannot activate NF-κB. IKKβ-dependent NF-κB activation has also been shown to be responsible for inhibition of NGF-promoted neurite growth from sympathetic neurons by TNFα [20]. By contrast, inhibition of BDNF-promoted neurite growth from nodose neurons by the TNFSF member LIGHT is because of a reduction of NF-κB transcriptional activity in these neurons [28]. In nodose neurons, tyrosine kinase-dependent NF-κB activation and dephosphorylation of p65 at ser536 contributes to BDNF-promoted neurite growth [29]. Thus, it appears that the TNFSF members that inhibit neurite growth may do so by modulating NF-κB signalling in different ways.

The TNF superfamily has been increasingly implicated in the regulation of neurite growth during development. In addition to the inhibitory effects of RANKL reported here, and the inhibitory effects of TNFα [20,30] and LIGHT [28], certain other members of the TNF superfamily enhance neurite growth. For example, FasL promotes neurite growth from DRG explants [31], and increases neurite branching from cultured hippocampal and cortical neurons [32]; and GITRL–GITR signalling enhances NGF-promoted neurite growth from SCG neurons [33,34]. TNF superfamily members exert their opposing effects on neurite growth from various kinds of neurons over differing periods of development. Whereas, for example, LIGHT and TNFα exert their respective inhibitory effects on sympathetic and sensory neurite growth over narrow developmental windows in the immediate perinatal period [20,28], RANKL inhibits the growth of sympathetic and sensory neurites over an extended period of post-natal development. Thus, a complex picture is emerging of multiple members of the TNF superfamily facilitating or inhibiting the growth and branching of the neural processes of particular kinds of neurons over distinctive periods of development. It is conceivable that the collective involvement of members of the TNF superfamily in regulating neurite growth during development form part of the array of extracellular signals that orchestrate the distinctive patterns of growth and arbourization of different subsets of axons required to establish appropriate patterns of connectivity during the normal course of development.

## 5. Material and methods

### 5.1. Real-time QPCR

The levels of RANK and RANKL mRNAs were quantified relative to a geometric mean of mRNAs for the house-keeping enzymes GAPDH and SDHA, in dissected tissue by RT-QPCR [35]. Total RNA was extracted from whole SCG and sympathetic target organs with the RNeasy Mini extraction kit (Qiagen) and 5 µl was reverse transcribed, for 1 h at 45°C, with the AffinityScript kit (Agilent) in a 25 µl reaction according to the manufacturer's instructions. A total of 2.0 µl cDNA was amplified in a 20 µl reaction volume using Brilliant III ultrafast QPCR master mix reagents (Agilent). QPCR products were detected using dual-labelled (FAM/BHQ1) hybridization probes specific to each of the cDNAs (MWG/Eurofins). The PCR primers were: RANKL forward, 5′**-**ATA CAT GTG TAA GAC TAC TAA GAG AC 3′ and reverse, 5′**-**AAT CTA ACA TCA CCT ATG GAC TTT AC **-**3′; RANK forward, 5′-TTA AAC TAT TGG CTG TCT A**-**3′ and reverse, 5′**-**AAC TCT ATT CAT TCT TTC TTG**-**3′; GAPDH forward, 5′-GAG AAA CCT GCC AAG TAT G-3′ and reverse, 5′-GGA GTT GCT GTT GAA GTC-3′; SDHA forward, 5′-GGA ACA CTC CAA AAA CAG-3′ and reverse, 5′-CCA CAG CAT CAA ATT CAT-3′. Dual-labelled probes were RANKL, FAM-CCC ACG GTG TAT GAA ACT CAC AGC C-BHQ1; RANK, FAM-ATT TCC CTG GCA CCT TCA TTT-BHQ1; GAPDH, FAM-AGA CAA CCT GGT CCT CAG TGT-BHQ1; SDHA, FAM-CCT GCG GCT TTC ACT TCT CT-BHQ1. Forward and reverse primers were used at a concentration of 150 nM each, and dual-labelled probes were used at a concentration of 300 nM. PCR was performed using the Mx3000P platform (Agilent) using the following conditions: 45 cycles of 95°C for 12 s and 60°C for 35 s. Standard curves were generated in every 96-well plate, for each cDNA for every real-time PCR run, by using serial threefold dilutions of reverse transcribed spleen total RNA (Ambion). The standard curves allowed a numerical value to be assigned to the levels of RANK, RANKL and reference cDNAs in each reverse transcribed sample. Four separate dissections were performed for each age.

### 5.2. Neuron culture

SCG, trigeminal and nodose ganglia dissected from CD1 mice were trypsinized and plated at very low density (approx. 500 neurons per dish) in polyornithine/laminin-coated 35 mm tissue culture dishes (Greiner) in serum-free Hams F14 medium [36] supplemented with 0.25 per cent Albumax I (Invitrogen). Neuronal survival was estimated by counting the number of attached neurons within a 12 × 12 mm grid in the centre of each dish 2 h after plating and again after 24 h, and expressing the 24 h count as a percentage of the 2 h count. Analysis of the size and complexity of neurite arbours was carried out 24 h after plating. The neurite arbours were labelled either by incubating the neurons with the fluorescent vital dye calcein AM (Invitrogen) at the end of the experiment or by transfecting the neurons with a YFP expression plasmid prior to plating. Transfection was carried out by electroporation using the Neon Transfection system (Invitrogen). Images of neurite arbours were acquired by fluorescence microscopy, and analysed to obtain total neurite length, number of branch points and Sholl profiles [37]. Sholl analysis was carried out at either 30 or 50 μm intervals.

To estimate the relative level of NF-κB transcriptional activity, neurons were transfected with a plasmid expressing GFP under the control of an NF-κB promoter, and cultured in well dishes of 9 mm diameter. Neurons were imaged with a Zeiss Axioplan confocal microscope 4 and 24 h after transfection, and the number of GFP-positive neurons per well was counted. All imaging and quantification was performed blind. Statistical comparisons were performed by ANOVA followed by Fisher's *post hoc* test.

For studying the effects of soluble RANKL on local neurite growth, SCG neurons were seeded in one compartment of a two-compartment microfluidic device (Xona Microfludics, CA, USA). The cell body compartment was supplemented with 10 ng ml^−1^ NGF to sustain neuronal survival, and the neurite compartment received either NGF alone or NGF plus RANKL. Calcein AM (Invitrogen) was added to the neurite compartment 24 h after plating to label neurites in this compartment and identify the cell bodies that project neurite into this compartment. Neurite length was quantified by a modification of a previously described method [38]. Briefly, using NIH ImageJ, a grid of vertical lines was traced with an interline interval of 200 μm. Total intersections between neurites and the grid were counted and normalized against the number of labelled somas in the cell body compartment. Average neurite length per projecting cell body was calculated using the formula *L* = *DI*/2, where *L* is the estimated length, *D* the interline interval and *I* the average number of intersections per projecting cell body. Measurements were independently carried out in 10 separate fields along the microfluidic barrier.

Purified recombinant NGF, BDNF and RANKL were obtained from R&D Systems. IKKα, IKKβ, S536A-p65 and IκBα plasmids were provided by Ron Hay (Dundee University).

### 5.3. Western blots

Neurons were plated at high density in polyornithine/laminin-coated 96-well plates (70 000 neurons per well). The neurons were incubated overnight in medium containing 10 ng ml^–1^ NGF before treating for different intervals with 10 ng/ml^−1^ RANKL. After treatment, the cells were lysed in RIPA buffer and insoluble debris was removed by centrifugation. Samples were transferred to polyvinylidene difluoride membranes using the Bio-Rad TransBlot. Membranes were blocked with 5 per cent BSA in phosphate-buffered saline (PBS) containing 0.1 per cent TWEEN 20 and incubated with either anti-p65 (1 : 1000; Cell Signalling) or anti-phospho-S536-p65 (1 : 1000; Cell Signalling) antibodies that were detected with peroxidase-linked secondary antibodies (1 : 2000; Promega) and ECL-plus (Amersham). Densitometry was carried out using the Gel-Pro Analyzer 32 program (Media Cybernetics, Rockville, MD).

### 5.4. Immunocytochemistry

Cultured neurons were fixed in ice-cold methanol for 10 mins, washed three times with PBS and blocked with 5 per cent BSA in PBS for 1 h at room temperature. The cells were incubated overnight at 4°C with anti-β-III tubulin (1 : 1000; Promega) and anti-RANK (5 g ml^−1^; R&D Systems) in 1 per cent BSA. After three washes, the cells were incubated in Alexa-Fluor-labelled secondary antibodies (Invitrogen, 1 : 500) for 1 h. Controls (secondary antibody alone) were included in all studies.

## 6. Acknowledgement

This work was supported by a grant from the Wellcome Trust (grant no. 085984) to A.M.D. We thank Ron Hay of Dundee University for the K44A-IKKα, K44A-IKKβ, S536A-p65 and S32A/S36A-IκBα plasmids.

## Supplementary Material

Spreadsheet 1

## Supplementary Material

Spreadsheet 2

## Supplementary Material

Spreadsheet 3

## Supplementary Material

Spreadsheet 4

## Supplementary Material

Spreadsheet 5

## Supplementary Material

Spreadsheet 6

## Supplementary Material

Spreadsheet 7
